# The Effect of Comorbid Attention-Deficit/Hyperactivity Disorder Symptoms on Face Memory in Children with Autism Spectrum Disorder: Insights from Transdiagnostic Profiles

**DOI:** 10.3390/brainsci11070859

**Published:** 2021-06-28

**Authors:** Qi Chen, Zengjian Wang, Bin Wan, Qingxin Chen, Kun Zhai, Yu Jin

**Affiliations:** 1 Department of Maternal and Child Health, School of Public Health, Sun Yat-Sen University, Guangzhou 510080, China; chenq326@mail2.sysu.edu.cn (Q.C.); wanb.psych@outlook.com (B.W.); chenqx36@mail2.sysu.edu.cn (Q.C.); zhaik@mail2.sysu.edu.cn (K.Z.); 2 School of Psychology, South China Normal University, Guangzhou 510631, China; 3 Key Laboratory of Brain, Cognition and Education Sciences, South China Normal University, Ministry of Education, Guangzhou 510631, China; 4 Center for Studies of Psychological Application, South China Normal University, Guangzhou 510631, China; 5 Guangdong Key Laboratory of Mental Health and Cognitive Science, South China Normal University, Guangzhou 510631, China; 6 Max Planck Institute for Human Cognitive and Brain Sciences, 04103 Leipzig, Germany

**Keywords:** autism spectrum disorder, attention-deficit/hyperactivity disorder, face memory, executive function, comorbidity, heterogeneity, transdiagnostic

## Abstract

Face memory impairments are common but heterogeneous in autism spectrum disorder (ASD), which may be influenced by co-occurrence with attention-deficit/hyperactivity disorder (ADHD). Here, we aimed to investigate the phenotype change of face memory in children with ASD comorbid ADHD symptoms, and discuss the potential role of executive function (EF). Ninety-eight children were analyzed in the present study, including ASD− (ASD-only, n = 24), ADHD (n = 23), ASD+ (with ADHD symptoms, n = 23) and neurotypical controls (NTC, n = 28). All participants completed two tests: face encoding and retrieving task and Wisconsin Card Sorting Test (WCST) for measuring face memory and EF, respectively. Results revealed that: compared with the NTC group, children with ASD− exhibited lower accuracy in both face encoding and retrieving, and participants with ASD+ showed lower accuracy only in the retrieving, whereas no differences were found among participants with ADHD. Moreover, in the ASD+ group, face encoding performance was correlated with response perseverative errors (RPE) and failure to maintain sets (FMS) of WCST; significantly, there were no group differences between ASD+ and NTC in these two indices. The transdiagnostic profiles indicated that comorbid ADHD symptoms could modulate the face encoding deficiency of ASD, which may be partially compensated by EF. Shared and distinct intervention strategies to improve social cognition are recommended for children undergoing treatment for each condition.

## 1. Introduction

Face memory impairment, being associated with social and cognition development, has been a candidate neurocognitive endophenotype of autism spectrum disorder (ASD) [1,2,3,4]. For example, better face memory performance of children with ASD could predict their lesser independent play and more cooperative interaction with peers [5]. Some scholars have even proposed that training on face memory is a promising method for improving social function in individuals with ASD [6]. Yet, the phenotypic manifestation of face memory in ASD remains insufficiently understood due to the striking heterogeneity [7,8,9].

ASD in children often co-occurs with symptoms of attention-deficit/hyperactivity disorder (ADHD), reaching approximately 1/2~2/3 [10,11], that pose serious challenges to clinical practice and research data [12]. Recent perspectives have proposed that, for children with mild or high-functioning ASD, the ADHD-comorbid condition may mask their cognitive profiles, camouflage their difficulties, and led to their atypical phenotype changes [13,14,15]. Both ASD and ADHD face social difficulties, but their presentations are different: children with ASD primarily present in absence of positive behaviors, demonstrating limited social play and initiating fewer social interactions [16,17,18]; while ADHD predominantly manifest in the presence of negative behaviors including disobeying rules, inappropriate responses to others, and aggressive motions [17,19,20]. These shared and unique behavioral features between ASD and ADHD may originate from their diverse underpinning neurocognitive mechanisms [17], such as face memory deficits.

The deficiency of face memory has been widely reported in the ASD population [2,21,22], however, there has been limited discussion of the detailed processing characteristics of them, and with mixed results. The successful use of memory depends on the manipulation of encoding, maintenance and retrieval [23]. Some findings suggest that the memory deficiency of ASD can be attributable to the anomalies in encoding, characterized by difficulties in abstracting stimuli’ properties [24,25,26], whereas others hold that the attenuated face memory may be caused by atypical retrieval, reflected by the barriers in recalling existing information [5,27]. Neuroimaging studies have found that children with ASD presented underconnectivity between the fusiform face area (FFA) and the medial and rostral lateral prefrontal cortex during face memory encoding and retrieving [28]. For children with ADHD, it is still under debate whether they have face memory impairment or not. Previous studies have prompted that the face memory ability of individuals with ADHD would be less affected, with great likelihood of face retrieval being impacted [29,30,31]. For instance, Kim et al. [30] found that there were no group differences between ADHD and NTC in behavioral performance of memory retrieving, but participants with ADHD demonstrated lower neural processing efficiency. Such transdiagnostic cognitive phenotyping may be essential for understanding shared mechanisms underlying ASD and ADHD. Meanwhile, it will induce more uncertainties to the phenotype changes in children with ASD comorbid ADHD.

Studies have revealed that children having ASD with comorbid ADHD symptoms often present more severer social difficulties [32,33]. The strong relationship found between face memory and social behaviors suggests that children with comorbid conditions may have poorer abilities in face memory. However, notably, youth with comorbid conditions may display compounded characteristics of both disorders, having negative social behaviors as well as lacking positive ones [34]. As some scholars have proposed, this behavioral pattern that alternates between ASD and ADHD may result in unpredictable and atypical changes leading to impaired social cognition [17,35]. A study on event-related potentials (ERP) associated with emotional face memory found that children with ASD showed reduced N170 amplitude during memory encoding, and children with ADHD demonstrated reduced N400 amplitude during retrieving, while those with a comorbid condition performed abnormally during both encoding and retrieving [31]. These findings provided another possibility that ADHD-traits may alter the cognitive process of a child with ASD, to resemble the performance of an individual with ADHD.

To understand the underlying mechanisms of cognitive impairment in ASD and ADHD, executive function (EF) is one among the most robust tools [36,37,38]. It was found that EF plays an important role in social competence of ASD and ADHD [39,40], as well as being implicated in visual memory [41,42]. Zinke et al. [43] found that the visual memory performance of children with ASD was correlated with their ability of planning. Van et al. [44] found that executive dysfunction, rather than decreased storage capacity of information, contributed to explain visual memory impairments in ADHD. Although the exact relationship between face memory and EF has not been studied, both clinical practice and scientific investigation have prompted that EF, like sustained attention and cognitive flexibility, may significantly function in processes involved in face memory. Moreover, transdiagnostic studies have shown that different components of EF could dissociate respective features of ASD and ADHD, especially in the neurocognitive phenotype [45,46,47,48]. In our former study, it was also found that diverse indices of EF measured by Wisconsin Card Sorting Test could explain visual memory performance of children with ASD and ADHD [49]. For children with a comorbid condition, their characteristics of EF were more affected by the ADHD-trait, irrespective formal diagnosis [35,50,51]. Therefore, it is significant to explore the potential role of EF on face memory in children with ASD and ADHD, and especially among children with comorbidity.

With a growing awareness of the complexity and significance of comorbidity in ADHD and ASD, an increasing number of studies have encouraged transdiagnostic approaches to explain their similarities and differences in etiology, associated impairments, and the interventions [13]. Thus, the present study had a three-folded purpose. First, to compare the transdiagnostic features of face memory between children with ASD and those with ADHD. Second, to further analyze the manifestation change in face memory among children with ASD comorbid ADHD symptoms. Third, to preliminary explore the correlation between face memory and EF.

## 2. Materials and Methods

The study procedure was approved by the medical ethics committee of the affiliated institute of the authors (consistent with the Declaration of Helsinki) prior to recruitment of participants. Written informed consent was obtained from the guardians. All participants did not take any central nervous system active medications in recent half years.

### 2.1. Participants

A convenience sample of 106 right-handed children, aged 6–12 years, was recruited through advertisement. The inclusion criteria were as follows: (1) having a full intellectual quotient (FIQ) score ≥70, with normal naked vision or corrected-vision without color blindness; (2) having the ability to speak and comprehend Mandarin; (3) having no history or presence of neurological or severe medical illness, such as learning disorder, schizophrenia, bipolar disorder, major depressive disorder, anxiety disorder, etc.; and (4) completing all the administered measures. In addition, children with NTC were required to have normal (not clinical or subclinical) scores on both rating scales of ASD and ADHD symptoms.

Children with ASD or ADHD were previously diagnosed by professional pediatricians in licensed hospitals in Guangzhou City or Shenzhen City, and the diagnosis certificates were obtained from the guardians. Their diagnosis were then re-confirmed by two pediatricians and psychiatrists experienced in ASD and ADHD assessment, according to the DSM-5 [52]. Based on their scores on the Swanson, Nolan, and Pelham-IV rating scales (SNAP-IV), participants with ASD were divided into two groups: ASD− group (ASD-only, without ADHD symptoms) and ASD+ group (with ADHD symptoms). Moreover, two well-trained researchers have completed the behavioral observation and interview with guardians to confirm whether children with ASD had co-occurring with ADHD symptoms. Children with NTC were recruited from primary schools.

The Chinese version of the Wechsler Intelligence Scale for Children, fourth edition (C-WISC-IV), was used to assess the cognitive profile of all participants, which included full intellectual quotient (FIQ) and four sub-indices: verbal comprehension index (VCI), perceptual reasoning index (PRI), working memory index (WMI), and processing speed index (PSI).

### 2.2. Clinical Characteristics

The Child Autism Rating Scale (CARS) [53] and Social Responsive Scale (SRS-2) [54] were applied to assess ASD symptoms. The Chinese version of the 18-items SNAP-IV [55] was used to measure ADHD symptoms. The CARS was measured by two well-trained researchers in the present study. In addition, parents of all participants were instructed to rate their children’s psychological and behavioral status according to their daily performance of the latest half years.

### 2.3. Face Memory

**Figure 1:**
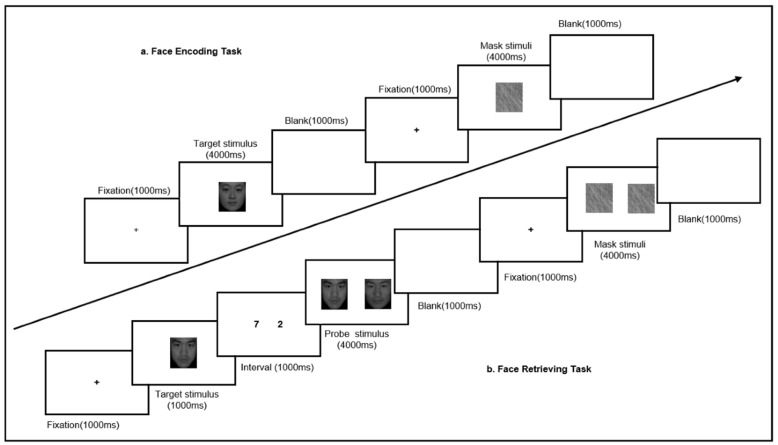
A demonstration of face encoding and retrieving paradigm. (**a**) Face encoding Task. (**b**) Face Retrieving Task.

The paradigm consisted of a face encoding task (FET) and a face retrieving task (FRT) to assess face memory performance (see Figure 1). Both tasks have been successfully applied in previous researches with different samples [56,57,58]. The participants were instructed to complete the FET first, which was followed by the FRT. The two sessions were divided by a short break of approximately 2 min.

*Face encoding task (FET)*: Each trial was preceded by a fixation (the character ‘+’) presented at the center of the screen for 1000 ms. Next, single pictures of neutral faces were presented for 4000 ms in a pseudo-randomized order, and participants were required to distinguish the gender of the facial images. They were instructed to press the ‘F’ button if the person was female and press the ‘J’ button if the person was male. Following this, the stimulus was replaced by a blank screen for another 1000 ms to complete the trial.

*Face retrieving task (FRT)*: Similar to FET, each trial of FRT began with a fixation for 1000 ms. This was followed by a target stimulus which was presented at the center of the screen for 1000 ms, and participants were asked to actively memorize each face. Next, two pictures—one previously presented and another new image—of faces were presented side by side for 4000 ms. Participants were requested to select the previously presented face by pressing the ‘F’ button if it appeared on the left, while pressing the ‘J’ button if it appeared on the right. During the interval between the target and probe stimulus (1000 ms), the participants were asked to read out the two numbers appearing on the screen. Similar to FET, the trial ended with a blank screen for another 1000 ms.

In addition, the mask blocks were used as the baseline condition. The stimulus were gray scrambled images which required participants to press the ‘J’ button within 4000 ms. Each task consisted of five blocks, including six trials of target stimulus and six mask stimuli, such that the FET was completed within 6 min and FRT was done within 7 min. There were two practice blocks before the actual task, to enable the participants to learn the rules. The mean response time (RT) and accuracy (ACC) were recorded.

The face images were selected from the Chinese Facial Affective Picture System. The height of the picture was 300 pixels and the width was 260 pixels. The paradigm was designed using E-prime 2.0 and displayed on a 20-inch LCD screen (1920 × 1080 pixels resolution). The visual stimuli were subtended at a visual angle of 0.5–1 degree in the horizontal plane at eye-level.

### 2.4. Executive Function

The Wisconsin Card Sorting Test (WCST) is a well-established measurement of EF [59]. Here, EF was examined by a computerized WCST which comprised of 128 reaction cards. The stimuli cards differed in colors, shapes, and numbers while the response card combined these factors to match the different stimuli cards based on diverse rules. This generated 11 familiar scores, and 4 indices were used in the present study: (1) categories completed (CC) reflecting the conceptual ability, (2) response errors (RE) reflecting the ability of switching, (3) response perseverative errors (RPE) revealing the ability of flexibility, and (4) failure to maintain sets (FMS) representing sustained attention.

### 2.5. Statistical Analysis

Data analysis was performed using the SPSS 25.0 software. First, multiple Shapiro–Wilk tests were conducted to inspect the distribution of quantitative variables, including age, score of CARS, score of SRS, score of SNAP-IV, score of WCST indices and the accuracy (ACC) and response time (RT) of face encoding and retrieving. Based on the results, parametric tests were employed for normally distributed variables and non-parametric tests were used for non-normally distributed variables (including PSI, CARS score, SRS subdomain scores, and indices of WCST).

Second, a one-way analysis of variance (ANOVA) or Kruskal–Wallis test was performed to determine the group differences in demographic characteristics, related symptoms, and face memory performance, and a Bonferroni correction was used for multiple comparisons. In addition, analysis of covariance (ANCOVA) was applied to compare face memory performance among the four groups after controlling for age and PSI/FIQ.

Third, a Pearson/Spearman correlation analysis was carried out to test the relationship between face encoding and retrieving ability and the symptoms of ASD and ADHD. Furthermore, multi-level linear regression analysis was employed to explore the relationship between EF and face memory performance. The size of test was set at *α* = 0.05.

## 3. Results

### 3.1. Sample Characteristics

Six participants including children with ASD− (n = 2), ADHD (n = 1), and ASD+ (n = 3) were excluded as they could not accomplish the behavioral tasks. Furthermore, an initial screening based on the omission rate of the mask-task was conducted to yield two outlier participants (*Z* score beyond ± 3 of the sample as a whole). Thus, a total of 98 participants (24 ASD−, 23 ADHD, 23 ASD+, 28 NTC) were included in the final analysis.

The sociodemographic and clinical information of the participants are shown in Table 1. There were no significant group differences in age (*F*_(3.94)_ = 2.602, *p* = 0.745) or sex distribution (*χ*^2^ = 2.048, *p* = 0.562). Instead, group differences were observed in the FIQ (*F*_(3.94)_ = 5.395, *p* = 0.002, *η*^2^ = 0.147) and PSI (*H*_(3.94)_ = 21.954, *p* < 0.001) scores. Furthermore, the post hoc test showed that the ASD+ group presented lower FIQ scores compared to the NTC group (*t* = −16.627, *p* = 0.001), and all clinical groups had lower PSI scores than NTC (all pairwise comparisons, *p* ≤ 0.005).

There were significant differences in the total score of CARS (*H*_(3.94)_ = 80.265, *p* < 0.001), SRS (*F*_(3.94)_ = 30.821, *p* < 0.001, *η*^2^ = 0.496), and SNAP-IV (*F*_(3.94)_ = 35.030, *p* < 0.001, *η*^2^ = 0.531) among the groups. In the scores on CARS, children with ASD− and ASD+ scored higher than those with ADHD and NTC (all pairwise comparisons, *p* ≤ 0.001), and children with ADHD scored higher than NTC (*z* = 21.659, *p* = 0.04). In SRS, the ASD+ group scored higher than the other three groups (all pairwise comparisons, *p* ≤ 0.001), and both ASD− and ADHD groups scored higher than NTC (ASD− vs. NTC, *t* = 23.804, *p* < 0.001; ADHD vs. NTC, *t* = 16.950, *p* = 0.013). In SNAP-IV, the post hoc test demonstrated that the ADHD and ASD+ groups scored higher than the ASD− and NTC groups (all pairwise comparisons, *p* ≤ 0.001). This reflected that the sample of the four groups was well-characterized in the clinical profile.

### 3.2. Ability of Face Memory

At first, the ANOVA results showed group differences of RT in the mask condition in both FET and FRT, which implied that the basic response level may be unequal for the four groups. To address this, an analysis of covariance (ANCOVA) was further conducted after controlling for age and PSI, and age and FIQ, respectively. Considering our former study which indicated that face memory performance would be affected by processing speed index of WISC-IV [60], and that the two results controlling PSI or FIQ were similar, in this context, the results of ANCOVA after controlling age and PSI are primarily reported. (see detailed in Table 2).

The ANCOVA results revealed that there were no significant interactions between group and age (*F*_(3.94)_ = 1.131, *p* = 0.341), nor between group and PSI (*F*_(3.94)_ = 1.215, *p* = 0.309). Notably, there were significant group differences in ACC of encoding (*F*_(3.94)_ = 5.145, *p* = 0.002, *η*^2^ = 0.144) and ACC of retrieving (*F*_(3.94)_ = 9.641, *p* < 0.001, *η*^2^ = 0.239). Multiple comparisons (using Bonferroni correction) indicated that children with ASD− exhibited a lower ACC than children with ADHD (*t* = −0.128, *p* = 0.026) and NTC (*t* = −0.159, *p* = 0.003) in FET; and in FRT, both children with ASD− and those with ASD+ displayed a lower ACC than those with ADHD and NTC (ASD− vs. ADHD, *t* = −0.197, *p*<0.001; ASD− vs. NTC, *t* = −0.172, *p* = 0.001; ASD+ vs. ADHD, *t* = −0.158, *p* = 0.003; ASD+ vs. NTC, *t =* −0.133, *p* = 0.023).

### 3.3. Correlation between Face Encoding and Retrieving and the Symptoms of ASD and ADHD

Table 3 shows the results of the Pearson correlation analysis (or Spearman analysis) between behavioral performance and the SRS and SNAP scores. In the ASD− group, RT-encoding was associated with SRS total score (*r* = −0.411, *p* = 0.046), social awareness (*r* = −0.462, *p* = 0.023), social cognition (*r* = −0.455, *p* = 0.025) and autistic behaviors (*r* = −0.418, *p* = 0.042), while ACC-retrieving (*r* = −0.418, *p* = 0.042) and RT-retrieving (*r* = −0.406, *p* = 0.049) were associated with social cognition. In contrast, in the ADHD group, only ACC-retrieving was found to be negatively correlated with social motivation (*r* = −0.428, *p* = 0.041). As for children with ASD+, ACC-encoding was associated with SRS scores, including total score (*r* = −0.433, *p* = 0.039), and social motivation (*r* = −0.521, *p* = 0.011), and RT-retrieving was associated with social awareness (*r* = −0.593, *p* = 0.003). Moreover, it was found that RT-encoding was correlated with SNAP total score (*r* = −0.497, *p* = 0.016) and the sub-index of hyperactivity (*r* = −0.550, *p* = 0.007). Additionally, a multiple linear regression controlling age and FIQ was conducted, and the results are demonstrated in Supplementary Table S1.

### 3.4. Association between Face Memory and Executive Function

In the comparison of EF, all clinical groups had lower score in CC compared to the NTC group (*H*_(3.94)_ = 18.781, *p* < 0.001; all pairwise comparison, *p* < 0.05); and participants with ADHD and those with ASD+ scored higher than NTC in the index of RE (*H*_(3.94)_ = 16.886, *p* = 0.001; ADHD vs. NTC, *p* = 0.001; ASD+ vs. NTC, *p* = 0.006). There were no group differences in RPE and FMS (see Table 4).

To explore the relationship between EF and performance of face encoding and retrieving performance, a multi-linear regression analysis (stepwise) adjusting for age and FIQ was employed. The results (see Table 5) illustrated that CC predicted 25.8% individual variation of ACC-retrieving in ASD− group (*F*_(1.22)_ = 7.660, *p* = 0.011, *R*^2^ = 0.258), and RPE predicted 26.8% individual difference of RT-retrieving in the ADHD group (*F*_(1.21)_ = 7.672, *p* = 0.011, *R*^2^ = 0.268). In the ASD+ group, RPE and FMS predicted 33.8% performance of RT-encoding (*F*_(1.20)_ = 5.104, *p* = 0.016, *R*^2^ = 0.338), and RPE predicted 35.3% performance of RT-retrieving (*F*_(1.21)_ = 11.438, *p* = 0.003, *R*^2^ = 0.353). None of the of the EF indices predicted the performance of face encoding and retrieving in the NTC group. A Spearman correlation analysis between face memory and EF was also conducted, and the results are demonstrated in Supplementary Table S2.

## 4. Discussion

On the basis of disclosing the transdiagnostic commonalities and differences between ASD and ADHD, this study has elucidated an interesting issue about the phenotype change of face memory in children with ASD comorbid ADHD symptoms. The findings revealed that, (1) children with ASD− exhibited lower accuracy than NTC in both face encoding and retrieving, and participants with ADHD behaved similar to NTC, while those with ASD+ performed poorer than NTC only in face retrieving; (2) the face memory performance of all clinical groups were correlated with social behaviors reflected by SRS scores, but children with ASD+ continued to show an association between face encoding and ADHD symptoms measured by SNAP scores; (3) diverse indices of EF assessed by WCST could partially explain the manifestation of face memory in children with ASD−, ADHD and ASD+, respectively.

### 4.1. Transdiagnostic Features of Face Memory in Children with ASD and ADHD

As we hypothesized, the current study replicated the well-documented findings that children with ASD have general face memory deficits [22,61,62], and further affirmed that deficits are present both in the encoding and retrieval phases. In the behavioral aspect, Gaigg et al. [25] found that children with ASD present a lower accuracy than NTC in a three-order load paradigm of memory encoding, while Cooper et al. [27] found that they made more errors in a continuous memory recalling task. In the aspect of neural mechanism, functional magnetic resonance imaging (fMRI) studies indicated that children with ASD have lower neural processing efficiency during face encoding and retrieving, respectively [28,63]. Furthermore, results of this study found that face memory performance of children with ASD correlated with their social behaviors, as most previous studies demonstrated [2,3,5]. Remarkably, the insight from twin studies indicated that the heritable influences on face memory competence were associated with the social deficit aspect of autistic trait [2,3,64]. Since visual encoding competence sets the stage for successful face memory, strategic learning of early face encoding may enhance face recognition in children with ASD, and could show promise to remediate their social skills.

Consistent with previous studies [21,49,65], our findings did not support the prevalence of face memory deficits in children with ADHD. In addition, face memory performance of participants with ADHD were also found to be correlated with social behaviors, while the severity of social dysfunction in ADHD was milder than those with ASD (reflected by lower scores on CARS and SRS). Therefore, individuals with ADHD may possess more knowledge of social skills, which is supported by better social cognition, such as with face memory [17]. Notably, being affected by the ADHD trait impacts the manifestation of social dysfunction in children with ADHD in a manner different from those with ASD. For example, children with ADHD are unable to concentrate their attention on the memory information and are distracted by their surroundings and other social cues, which increases their social difficulties [17,66]. Thus, the effect of ADHD-traits should not be neglected when discussing the relationship between face memory and social function in the ADHD population.

The above findings indicated that the features of face memory could differentiate ASD and ADHD to be the transdiagnostic cognitive phenotype. Several causes could be responsible for their diverse behavioral presentation, and the distinct anomalous social visual attention pattern is a relevant issue yet to be explored in depth [67]. Patients with ASD have shown local processing preference on encoding visual information, and had difficulty in adopting chunking strategies to integrate the visual details [68,69,70]. Meanwhile, meta-analysis indicated that using a visual reminder, as a form of compensatory processing, may help to improve memory encoding and further enhance visual memory competence [71]. The autistic-like processing style seemingly suggested that face memory has already been impaired in the early encoding stage. On the contrary, individuals with ADHD failed to abstract targeted visual information when facing multiple stimuli or stimuli with complex details [66,72]. Accordingly, it was inferred that this ADHD-like processing style would be characterized by relatively intact ability in memory encoding but with some difficulties in retrieval.

### 4.2. Characteristic of Face Memory in Children with ASD Comorbid ADHD Symptoms

While there were prone to more severe social dysfunction, participants with ASD comorbid ADHD did not demonstrate poorer performance in face memory. The behavioral data suggested that children with the comorbid condition may present an additive effect: they performed as well as NTC in the face encoding task (like participants with ADHD), and made more errors in the face retrieving (similar to participants with ASD). This indicated that co-occurrence with ADHD symptoms would make the cognitive profile of ASD unpredictable, rather than having a simple effect of aggravation or alleviation of preexisting deficits. Regarding face memory phenotype change in children with ASD comorbid ADHD symptoms, some speculations could be made, which are presented below.

First, being affected by the ADHD trait, children with ASD comorbid ADHD symptoms may adopt an ADHD-like processing pattern during face encoding. Correlation analysis showed that the face encoding competence of the ASD+ group was associated with ADHD symptoms except for the established relationship between face memory and social function. As previous studies have emphasized the predominant role of attention condition, such as with attention allocation and distractibility in memory processing of both ASD and ADHD [21,73,74], an ADHD-like cognitive style would change the local processing preference of children with ASD. More significantly, the “additive model” has hypothesized that individuals with the comorbid condition are likely to manifest the separate characteristic of ASD and ADHD, to exhibit a confluence of the two [75].

Second, the phenomenon of mismatch between social dysfunction and face memory deficiency in children with the comorbid condition could be associated with the cognitive compensation mechanism. Recently, some scholars have pointed out that some patients with ASD may “compensate” for their underlying difficulties: in order to navigate the challenges associated with their core symptoms, they may mobilize more other cognitive resources such as EF and IQ [76,77,78]. Livingston et al. [77] conducted the first experimental study to confirm that some children with ASD could compensate their deficiency of social cognition by relatively improved utilization of EF. Furthermore, neuroimaging studies have found that children with ASD demonstrated increased hippocampal recruitment in memory encoding, and exhibited more activation within multiple prefrontal cortex areas when viewing faces, which were believed to be mechanisms of compensatory neural processing [79,80]. Although these findings are not sufficient to establish any conclusion, the studies provide interesting and significant opinions that require further exploration.

### 4.3. Relationship between EF and Face Memory

Importantly, recent perspectives have proposed that the special relationship between EF and social cognition deficits could explain the co-occurrence of ADHD symptoms in ASD [81]. Moreover, EF may impact social competence by facilitating higher-order cognition such as visual memory processing [39]. As part of a preliminary exploration, the present study found that different indices of WCST representing EF could explain the face retrieval performance in ASD−, ADHD, and ASD+ group, respectively. Consistently, previous studies have illustrated the close relationship between EF and visual memory in both ASD and ADHD populations [41,43,44,82]. Our former study had also demonstrated that varying components of EF could be dissociated with visual memory in ASD and ADHD [49]. Thus, the distinct features of EF may contribute to different visual processing patterns in children with ASD and ADHD, and result in their differing behavioral performance of face memory.

Of further interest, some scholars suggested that the ability of EF may have an important compensation function in patients with ASD [77,78]. In the present study, children with ASD comorbid ADHD symptoms also showed a correlation between face encoding and EF, including FMS and RPE in the WCST. While children with the comorbid condition scored as well as participants with NTC in these two indices. It is posited that children with the comorbid condition may mobilize their limited but relatively intact EF components to compensate for their face encoding deficits. In a pilot cognitive intervention research, Macoun et al. [83] found that visual memory competence of children with ASD would be remediated substantially through the compensatory training of EF. This shows promise in the development of intervention methods for improving social cognition. However, participants with ASD and ADHD present different features on EF [45,46,50], and children with a comorbidity were more affected by the ADHD trait [35,50]. Thus, shared and distinct interventional approaches would be necessary for each condition.

### 4.4. Limitations and Future Directions

The present study has some limitations. First, the age span across our participants (6.02~12.64 years old) was considered too wide, and the intelligence level of ASD+ and NTC groups were mismatched. Thus, age and PSI/FIQ were included as covariance. Further studies are warranted to test the effects of age. Second, although the data dissociated the different phases of face memory, studies combined with ERP may better isolate the neural responses of encoding and retrieval. Accordingly, these findings should not be overinterpreted, and more precise experimental conditions and large sample sizes would increase the strength of evidence. Third, the measurement of WCST could not precisely discriminate the diverse components of EF, and WCST only measures the cool EF. Considering the significance of EF on the underlying mechanisms of neurocognitive changes in ASD, the relationship between EF and face memory should be thoroughly explored.

## 5. Conclusions

The present study confirmed that face memory in children with ASD was impaired in both the encoding and retrieving phases. Importantly, the study provides a new perspective and empirical evidence to show that co-occurring ADHD symptoms could modulate the face encoding deficits of ASD, which may be associated with EF. These findings, delineating the transdiagnostic profiles among participants with ASD, ADHD, and their comorbidity, propose that shared and distinct intervention approaches may be needed for children with each of the disorders, to improve social cognition. This study sheds light on the transdiagnostic neurocognitive basis of the conditions and emphasizes the importance of considering ADHD comorbidity in ASD when recommending treatment plans.

## Figures and Tables

**Table 1:** Demographic characteristics of the participants (n = 98).

	ASD−	ADHD	ASD+	NTC	*F/H*	*p*	Post hoc
Male (%)	21 (87.5%)	21 (91.3%)	20 (87%)	22 (78.6%)	1.708	0.662	
Age	7.8 ± 1.8	8.3 ± 1.4	7.9 ± 1.8	8.2 ± 1.8	0.412	0.745	
FIQ	97.8 ± 17.7	98.6 ± 15.8	92.1 ± 17.4	108.7 ± 9.5	5.395	0.002	3 < 4
VCI	97.2 ± 16.4	103.7 ± 13.5	93.1 ± 18.3	111.3 ± 10.5	7.429	<0.001	1, 3 < 4
PRI	104.1 ± 18.2	104.0 ± 17.6	98.4 ± 17.5	110.2 ± 10.9	2.276	0.085	-
WMI	100.8 ± 18.7	95.4 ± 14.2	94.8 ± 15.2	101.8 ± 13.9	5.536	0.136	-
PSI	87.5 ± 13.6 ^	88.6 ± 14.1	86.8 ± 19.2 ^	100.7 ± 9.8	21.954	<0.001	1, 2, 3 < 4
CARS #	31.4 ± 2.2	20.0 ± 1.8	31.4 ± 2.6	16.7 ± 1.5	80.265	<0.001	1, 3 > 2 > 4
SRS							
Total score	69.4 ± 22.1	62.5 ± 18.2	96.8 ± 18.7	45.6 ± 17.4	30.821	<0.001	3 > 1, 2 > 4
Awareness	9.5 ± 2.9	9.8 ± 2.5	12.4 ± 2.2	8.0 ± 1.9	14.148	<0.001	3 > 1, 2, 4
Cognition	13.1 ± 4.8	12.8 ± 4.2	19.6 ± 4.1	8.8 ± 4.2	26.515	<0.001	3 > 1, 2 > 4
Communication	23.9 ± 8.7	19.8 ± 6.9	31.5 ± 7.7	13.4 ± 7.3 ^	24.698	<0.001	3 > 1, 2 > 4
Motivation	10.4 ± 3.4	9.9 ± 3.4 ^	13.5 ± 4.5 ^	9.4 ± 4.3 ^	5.171	0.002	3 > 2, 4
Autistic behavior	12.4 ± 6.3	10.1 ± 5.9	19.8 ± 5.5	6.0 ± 3.2	30.107	<0.001	3 > 1, 2 > 4
SNAP							
Total score	36.3 ± 5.1	46.7 ± 6.6	47.8 ± 6.6	33.0 ± 6.6	35.030	<0.001	2, 3 > 1, 4
Inattention	20.5 ± 2.7	26.0 ± 4.3	26.1 ± 2.1	18.7 ± 3.9	31.226	<0.001	2, 3 > 1, 4
Hyperactivity	15.2 ± 3.7	20.7 ± 4.5	21.7 ± 5.9	14.3 ± 3.6	16.232	<0.001	2, 3 > 1, 4

Note: # variance inequality; ^ distributed non-normally; ASD−, autism spectrum disorder (without ADHD symptoms); ADHD, attention-deficit/hyperactivity disorder; ASD+, ASD children with ADHD symptoms; NTC, neurotypical controls; FIQ, full intellectual quotient; VCI, verbal comprehensive index; PRI, perceptual reasoning index; WMI, working memory index; PSI, processing speed index; 1, ASD− group; 2, ADHD group; 3, ASD+ group; 4, NTC group.

**Table 2:** Group differences in face encoding and retrieving performance.

	ASD−	ADHD	ASD+	NTC	*F*_1_	*P*_1_	*F*_2_	*P*_2_	*F*_3_	*P*_3_	Post hoc
FET											
ACC	0.61 ± 0.16	0.74 ± 0.20	0.67 ± 0.14	0.81 ± 0.10	7.928	<0.001	5.145	0.002	5.323	0.002	1 < 2, 4
RT	1547.13 ± 359.04	1377.73 ± 239.38	1532.99 ± 437.97	1274.72 ± 283.12	3.868	0.012	2.024	0.116	2.775	0.046	-
RT-mask	813.82 ± 207.01	678.47 ± 129.67	862.73 ± 245.45	648.71 ± 243.09	5.442	0.002	2.998	0.035	3.362	0.022	-
FRT											
ACC	0.60 ± 0.17	0.81 ± 0.14	0.64 ± 0.16	0.81 ± 0.17	12.118	<0.001	9.641	<0.001	9.131	<0.001	1, 3 < 2, 4
RT	1283.16 ± 457.58	1263.17 ± 312.42	1175.17 ± 421.23	1058.83 ± 280.75	1.973	0.123	1.780	0.156	1.920	0.059	-
RT-mask	580.81 ± 173.31	539.03 ± 159.50	554.92 ± 143.52	485.75 ± 135.74	1.808	0.151	0.659	0.580	0.785	0.505	-

Note: *F*_1_, *P*_1_: the results of ANOVA; *F*_2_, *P*_2_, and post hoc: the results of ANCOVA controlling for age and PSI; *F*_3_, *P*_3_: the results of ANCOVA controlling for age and FIQ; FET, face encoding task; FRT, face retrieving task; 1, ASD− group; 2, ADHD group; 3, ASD+ group; 4, NTC group; ACC, accuracy; RT, response time.

**Table 3:** Correlation between face memory performance and symptoms of ASD and ADHD.

	ASD− (n = 24)				ADHD (n = 23)				ASD+ (n = 23)			
	Encoding		Retrieving		Encoding		Retrieving		Encoding		Retrieving	
	ACC	RT	ACC	RT	ACC	RT	ACC	RT	ACC	RT	ACC	RT
**SRS**												
Total score	−0.271	−0.411 *	−0.219	−0.366	−0.124	0.012	−0.278	0.231	−0.433 *	−0.108	−0.403	−0.313
Awareness	−0.154	−0.462 *	0.045	−0.245	0.036	−0.273	−0.135	0.196	−0.129	−0.314	0.151	−0.593 **
Cognition	−0.339	−0.455 *	−0.418 *	−0.406 *	−0.118	−0.032	−0.061	0.101	−0.372	0.000	−0.317	−0.243
Communication	−0.194	−0.294	−0.227	−0.324	−0.072	0.000	−0.263	0.174	−0.286	−0.183	−0.395	−0.210
Motivation	−0.202	−0.107	−0.033	−0.199	0.100	0.132	−0.428 *	0.412	−0.521 *	0.210	−0.382	−0.230
Autistic behavior	−0.244	−0.418 *	−0.138	−0.307	0.290	0.097	−0.200	0.113	−0.337	−0.157	−0.331	−0.164
**SNAP**												
Total score	−0.266	−0.199	−0.254	−0.260	−0.345	0.129	−0.077	0.423	0.167	−0.497 *	0.160	−0.389
Inattention	−0.089	−0.131	−0.317	−0.295	−0.280	0.016	0.014	0.298	0.317	−0.002	−0.011	−0.197
Hyperactivity	−0.307	−0.183	−0.122	−0.147	−0.239	0.174	−0.127	0.337	0.071	−0.550 **	0.181	−0.360

Note: * *p* < 0.05; ** *p* < 0.01; ASD−, autism spectrum disorder (without ADHD symptoms); ADHD, attention-deficit/hyperactivity disorder; ASD+, ASD children with ADHD symptoms; ACC, accuracy; RT, response time; SRS, social response scale; SNAP, Swanson, Nolan, and Pelham-IV rating scales.

**Table 4:** Group differences of WCST indices.

	ASD−	ADHD	ASD+	NTC	*H*	*p*	Post hoc
CC	4 (2.6)	2 (2.6)	2 (2.6)	5 (2.6)	18.781	<0.001	1, 2, 3 < 4
RE	57 (16.82)	75.5 (16.107)	78 (27.95)	42 (9.107)	16.886	0.001	2, 3 < 4
RPE	6 (1.11)	6 (2.12)	4 (1.37)	7 (2.30)	1.753	0.188	-
FMS	2 (0.4)	1 (0.4)	1 (0.3)	1 (0.5)	2.158	0.540	-

Note: CC, categories completed; RE, response errors; RPE, response perseverative errors; FMS, failure to maintain sets; 1, ASD− group; 2, ADHD group; 3, ASD+ group; 4, NTC group.

**Table 5:** Multiple regression analysis between EF and VSWM across groups.

Group	Dependent	Independent	*β* (SE)	*b’*	*t*	*p*	*R* ^2^
ASD−	ACC-Retrieving	CC	0.043 (0.016)	0.508	2.768	0.011	0.285
ADHD	RT-Retrieving	RPE	−40.781 (14.724)	−0.517	−2.770	0.011	0.268
ASD+	RT-Encoding	FMS	163.741 (72.155)	0.414	2.269	0.034	0.338
		RPE	15.541 (7.435)	0.381	2.090	0.050	
	RT-Retrieving	RPE	23.284 (6.855)	0.594	3.382	0.003	0.353

Note: ASD−, autism spectrum disorder (without ADHD symptoms); ADHD, attention-deficit/hyperactivity disorder; ASD+, ASD children with ADHD symptoms; CC, categories completed; RE, errors responses; RPE, perseverative responses errors; FMS, failure to maintain set; ACC, accuracy; RT, response time.

## Data Availability

The datasets used and/or analyzed during the current study are available from the corresponding author on reasonable request.

## Supplementary Materials

The following are available online at https://www.mdpi.com/article/10.3390/brainsci11070859/s1, Additional statement: The diagnosis procedure of ASD and ADHD. Table S1. Multiple regression analysis between EF and VSWM in different groups. Table S2. The correlation between face memory performance and symptoms of ASD and ADHD.
