# Supplementary material for: The Effect of Comorbid Attention-Deficit/Hyperactivity Disorder Symptoms on Face Memory in Children with Autism Spectrum Disorder: Insights from Transdiagnostic Profiles
Source: Brain Sci. 2021 Jun 28;11(7):859. doi: 10.3390/brainsci11070859 (PMC8301798; doi:10.3390/brainsci11070859)
Supplement: Supplementary file 1 [file brainsci-11-00859-s001.zip › brainsci-1245946-supplementary.pdf]

Supplementary information

Additional statement: The diagnosis procedure of ASD and ADHD

First, all children with ASD and those with ADHD were previously diagnosed by professional pediatricians in licensed hospitals, such as Child Development &Behavioral Center of the Third Affiliated Hospital of Sun Yat-Sen University, Guangzhou Women and Children Medical Care Center, Shenzhen Children’s Hospital, etc.. Guardians were required to provide the diagnosis certificates.

Second, since the Autism Diagnostic Observation Schedule (ADOS) and the Autism Diagnostic Interview-Revised (ADI-R) have not been officially validated and widely used in China, we assessed the clinical symptoms of ASD and ADHD using Chinese version of CARS, SRS-2, and SNAP-IV among all participants. The CARS, together with some other assessment such as “false belief” test, were conducted by two pediatricians and psychiatrists experienced in ASD and ADHD assessment (Dr. Y.J. and Dr.J.J., director of our research center). They have observed the participants’ behavior during the assessment, and then interviewed with guardians to acquire more information about children’s daily performance at home and/or at school. Combining these information, we have discussed and re-confirmed the diagnosis of ASD and ADHD according to the DSM-5.

Table S1. Multiple regression analysis between EF and VSWM in different groups

Group	Dependent	Independent	*β* (SE)	*b'*	*t*	*P*	R^2
**ASD-**	**RT-encoding**	**SRS-awareness**	-57.41（23.5）	-0.462	-2.443	0.023	0.213
	**ACC-retrieving**	**SRS-cognition**	-0.02（0.01）	-0.418	-2.157	0.042	0.175
	**RT-retrieving**	**SRS-cognition**	-38.31（18.32）	-0.406	-2.084	0.049	0.165
**ADHD**	**ACC-retrieving**	**SRS-motivation**	-0.02（0.01）	-0.428	-2.172	0.041	0.183
	**RT-retrieving**	**SNAP-total score**	20.04（8.55）	0.423	2.344	0.030	0.349
		**SRS-motivation**	37.40（16.37）	0.412	2.285	0.033	
**ASD+**	**ACC-encoding**	**SRS-motivation**	-0.02（0.01）	-0.500	-2.710	0.013	0.342
		**SNAP-inattention**	0.03（0.01）	0.407	2.208	0.039	
	**RT-encoding**	**SNAP-hyperactivity**	-40.81（13.51）	-0.550	-3.019	0.007	0.303
	**RT-Retrieving**	**SRS-awareness**	-114.31（33.83）	-0.593	-3.379	0.003	0.352

Note: ASD-, autism spectrum disorder (without ADHD symptoms); ADHD, attention-deficit/hyperactivity disorder; ASD+, ASD children with ADHD symptoms; ACC, accuracy; RT, response time; SRS, scores of Social Response Scale; SNAP, scores of Swanson, Nolan, and Pelham-IV rating scales.

Table S2. The correlation between face memory performance and symptoms of ASD and ADHD.

	ASD- (n=24)				ADHD (n=23)				ASD+ (n=23)			
	Encoding		Retrieving		Encoding		Retrieving		Encoding		Retrieving	
	ACC	RT	ACC	RT	ACC	RT	ACC	RT	ACC	RT	ACC	RT
**CC**	0.111	0.110	**0.444***	0.320	0.268	**-0.351#**	0.222	**-0.501***	0.128	**0.546****	0.076	0.271
**RE**	-0.028	0.028	-0.301	-0.269	-0.286	0.104	-0.204	**0.425***	-0.112	**-0.358#**	-0.075	-0.168
**RPE**	-0.168	-0.072	0.113	0.192	-0.033	-0.319	-0.084	**-0.583***	0.301	0.300	-0.196	**0.436#**
**FMS**	-0.121	-0.304	0.055	0.174	-0.026	-0.183	0.305	-0.239	0.280	**0.463***	0.030	0.074

Note: *p<0.05; #p<0.1; CC, categories completed; RE, response errors; RPE, response perseverative errors; FMS, failure to maintain sets; ACC, accuracy; RT, response time.
